# Fixation of Paediatric Proximal Humerus Fractures Using Percutaneous Pinning: A Case Report

**DOI:** 10.7759/cureus.64888

**Published:** 2024-07-19

**Authors:** Abhishek Nair, Ashwin Deshmukh, Swaroop Solunke, Shubhankar Chopra, Archit Gupta

**Affiliations:** 1 Orthopaedics, Dr. D. Y. Patil Medical College, Hospital and Research Centre, Dr. D. Y. Patil Vidyapeeth, Pune (Deemed to be University), Pune, IND

**Keywords:** closed reduction, kirschner wires, percutaneous pinning, proximal humerus fracture, pediatric orthopedics

## Abstract

A 12-year-old male came to our Emergency Department with chief complaints of pain and inability to move the right shoulder for one day following a fall while playing. The range of motion of the right shoulder was restricted and painful in all directions. Initial radiographs revealed a transverse, displaced proximal humerus fracture at the head-shaft junction. The patient was managed by closed reduction internal fixation with percutaneous K-wiring (Kirschner wires). The K-wires were removed after four weeks, and the shoulder was mobilized. The patient had a near-normal and pain-free range of motion at three months of follow-up. Percutaneous K-wiring remains a viable option for the treatment of paediatric proximal humerus fractures, and good post-operative rehabilitation can help restore near-normal function, as demonstrated in this report.

## Introduction

Proximal humerus fractures in the paediatric age group comprise around 0.45-2% of all paediatric fractures [1,2]. The mechanism of injury is through blunt or indirect trauma causing either a displaced proximal fragment with the limb being adducted and externally rotated, or a displaced distal fragment with the limb in adduction, with apparent shortening [3].

Comprehension of the anatomy of the proximal humerus is a salient factor in management, to help in the reduction, and after that, remodelling. A universal standard classification for proximal humerus fractures is followed to aid treatment based on type, which is the Neer-Horwitz classification for paediatric proximal humerus fractures [4].

Treatment modalities can be non-operative and operative. Non-operative techniques include the use of sling, shoulder immobilization, or coaptation splint. Operative techniques include closed reduction (if unacceptable alignment but reduction is still possible), or open reduction with internal fixation (indicated when there is unacceptable reduction, open fractures, or vascular injuries) [5]. This case study aims to see the benefit of percutaneous pinning for proximal humerus fracture.

## Case presentation

A 12-year-old male came to our Emergency Department with chief complaints of pain over the right shoulder and inability to move the right shoulder for one day, following a fall while playing in the park. There were no other associated injuries. On examination, swelling was present; no open wound or abrasion was present over the right shoulder. On palpation, there was diffuse tenderness present. The range of motion of the right shoulder was restricted and painful. There were no associated comorbidities.

Pre-operative evaluation

**Figure 1:**
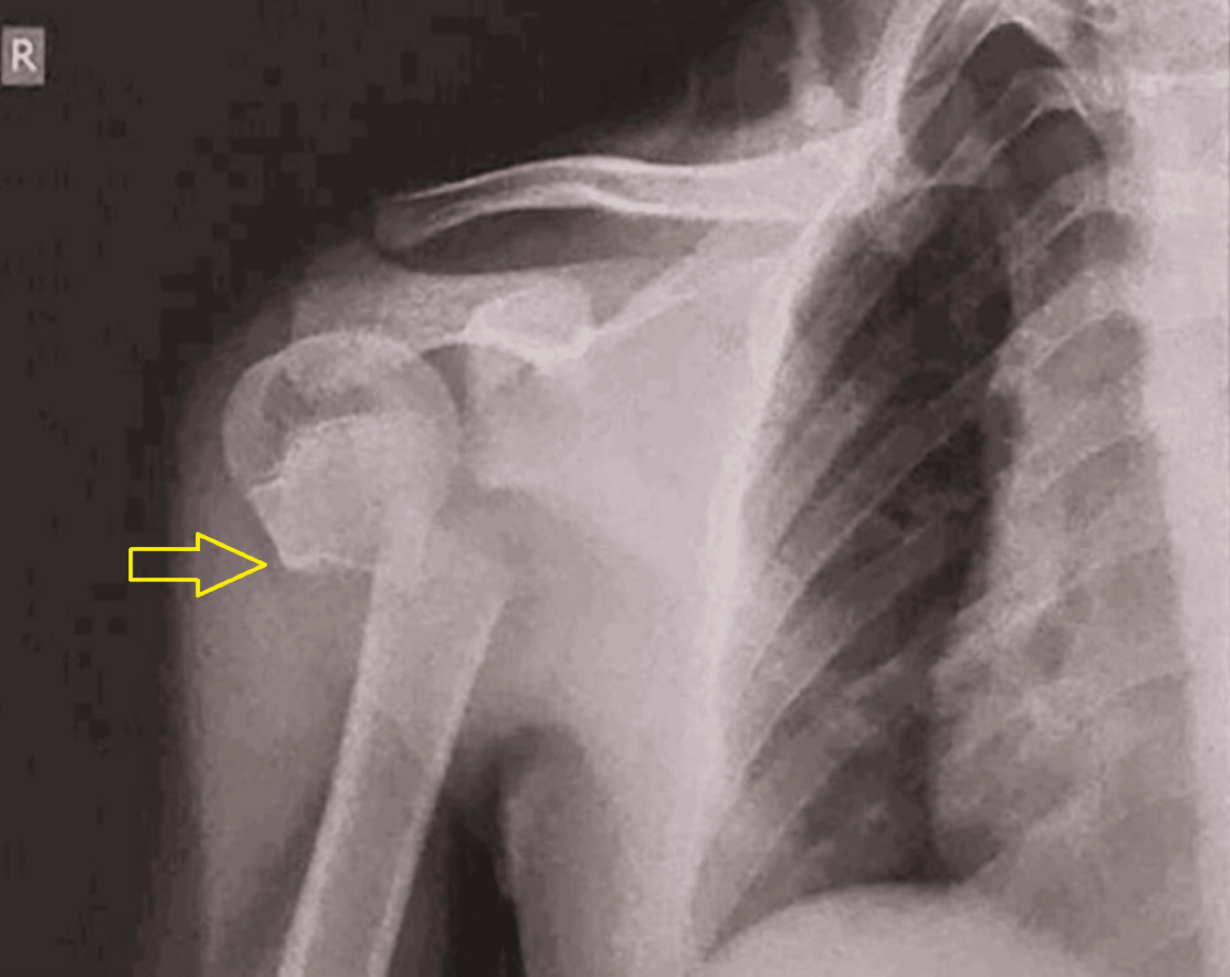
Anteroposterior view radiograph of the right shoulder showing proximal humerus fracture The yellow arrow shows the fracture site and displaced distal fragment.

Initial radiological workup revealed a transverse fracture of the right proximal humerus at the junction of the humeral head and shaft, sparing the physis, with the distal fragment displaced medially and superiorly (Figure 1). We classified the fracture as grade 4, according to Neer-Horwitz's classification of paediatric proximal humerus fracture [4]. 

Intra-operative findings

The patient was placed in a supine position with the head end of the table raised to 30°. The patient was positioned at the edge of the table, with his right arm outside the table for better accessibility and to create a better workable field.

**Figure 2:**
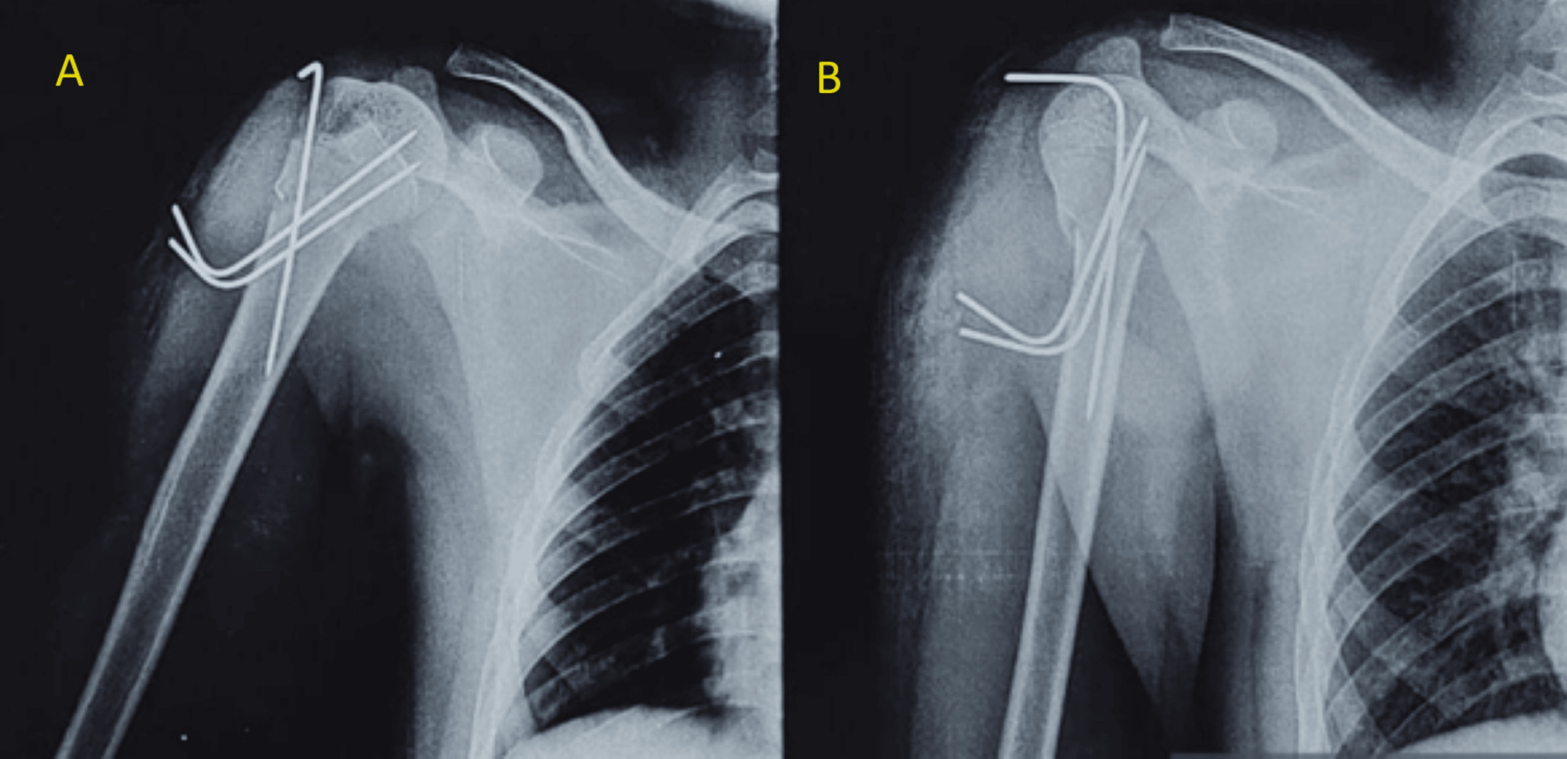
Radiograph anteroposterior view (A) and lateral view (B) of the right humerus with Kirschner wires in situ

Proximal humerus fracture was first reduced coherently by keeping a folded sterile drape under the axilla, and concurrent traction was applied along with adduction of the arm. Successful fracture reduction was achieved, corroborated by fluoroscopy. A 2.5 mm Kirschner wire (K-wire) was inserted from the lateral aspect of the humeral head in a superolateral to inferomedial direction into the shaft. Afterwards, two 2.5 mm K-wires, making a convergent angle with the initial K-wire, were inserted parallel to each other from the lateral aspect of the proximal humerus shaft in the inferolateral to the superomedial direction towards the humeral head. The placement of the three K-wires was checked again through fluoroscopy before cutting and bending the wires (Figure 2). 

Post-operative follow-up

At Four Weeks Follow-Up

**Figure 3:**
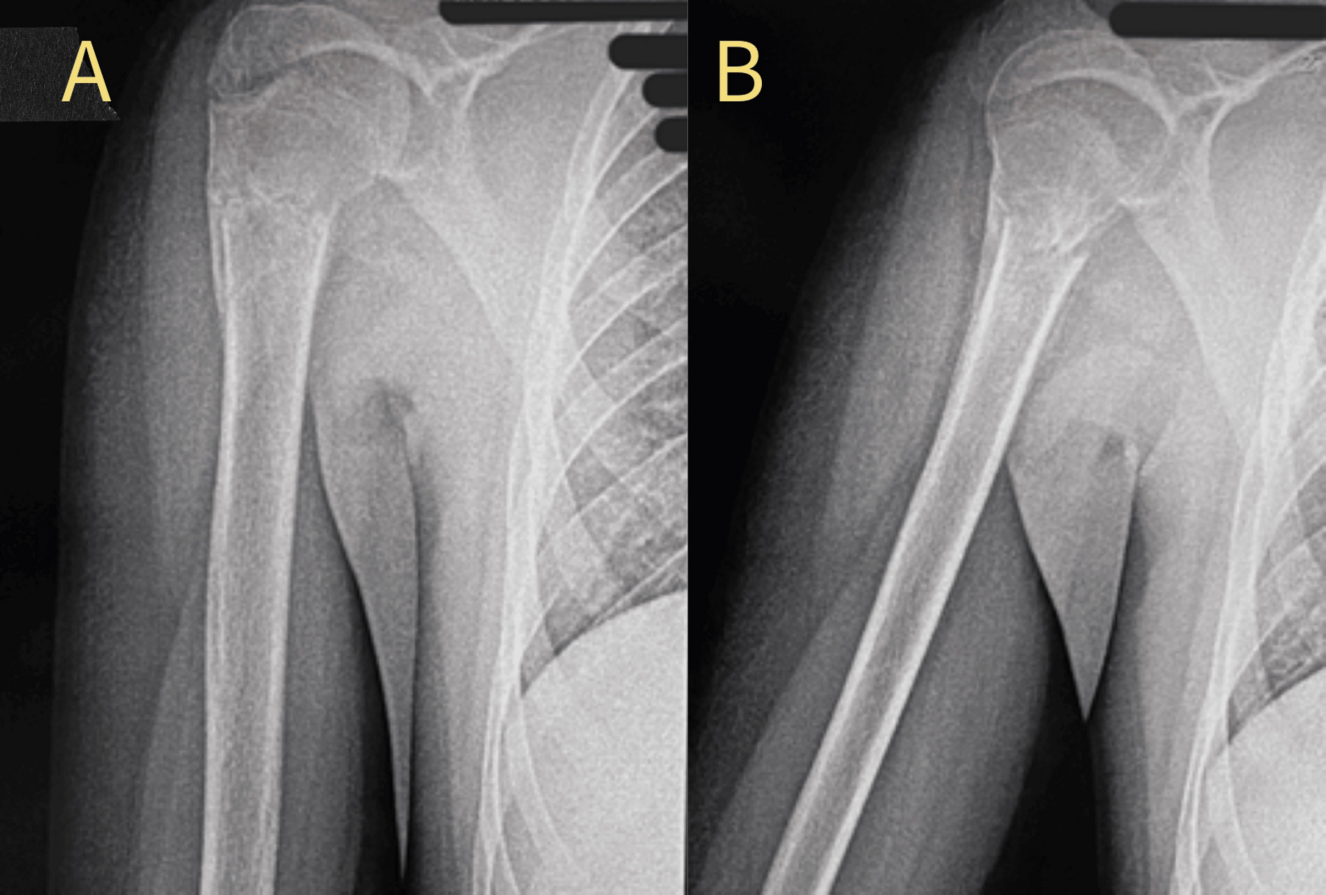
Radiograph at four weeks follow up showing (A) anteroposterior view and (B) lateral view of the proximal humerus after removal of the K-wires K-wire: Kirschner wire

Immediate post-op, a shoulder sling was given, and the patient was kept immobilized for four weeks. At the four-week follow-up, K-wires were removed (Figure 3), after which passive and active assisted movements were started. Active (unassisted) range of motion exercises began at the six-week follow-up.

At Three Months Follow-Up

**Figure 4:**
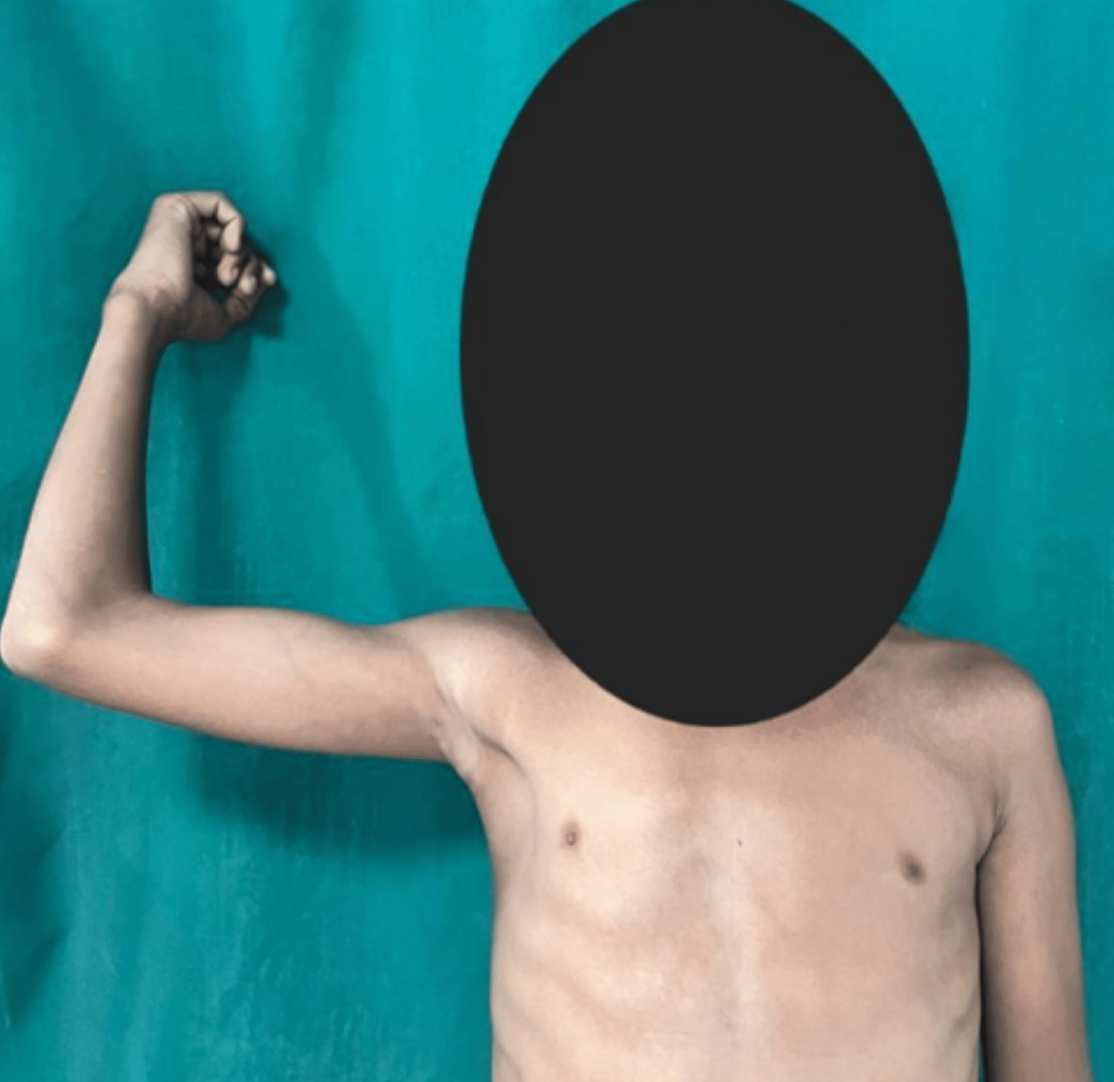
External rotation of the right shoulder in 90° elbow flexion

**Figure 5:**
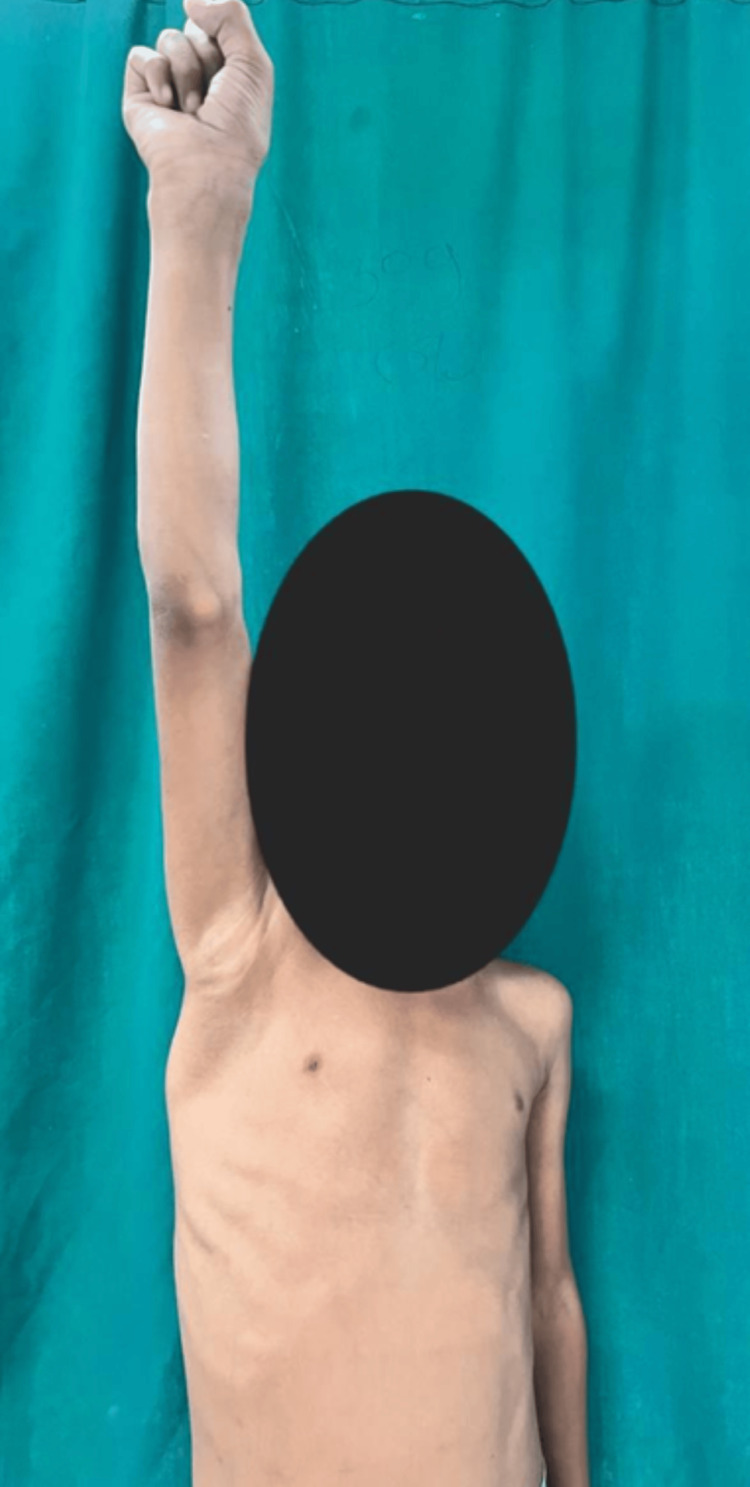
Abduction/forward flexion of the right shoulder

**Figure 6:**
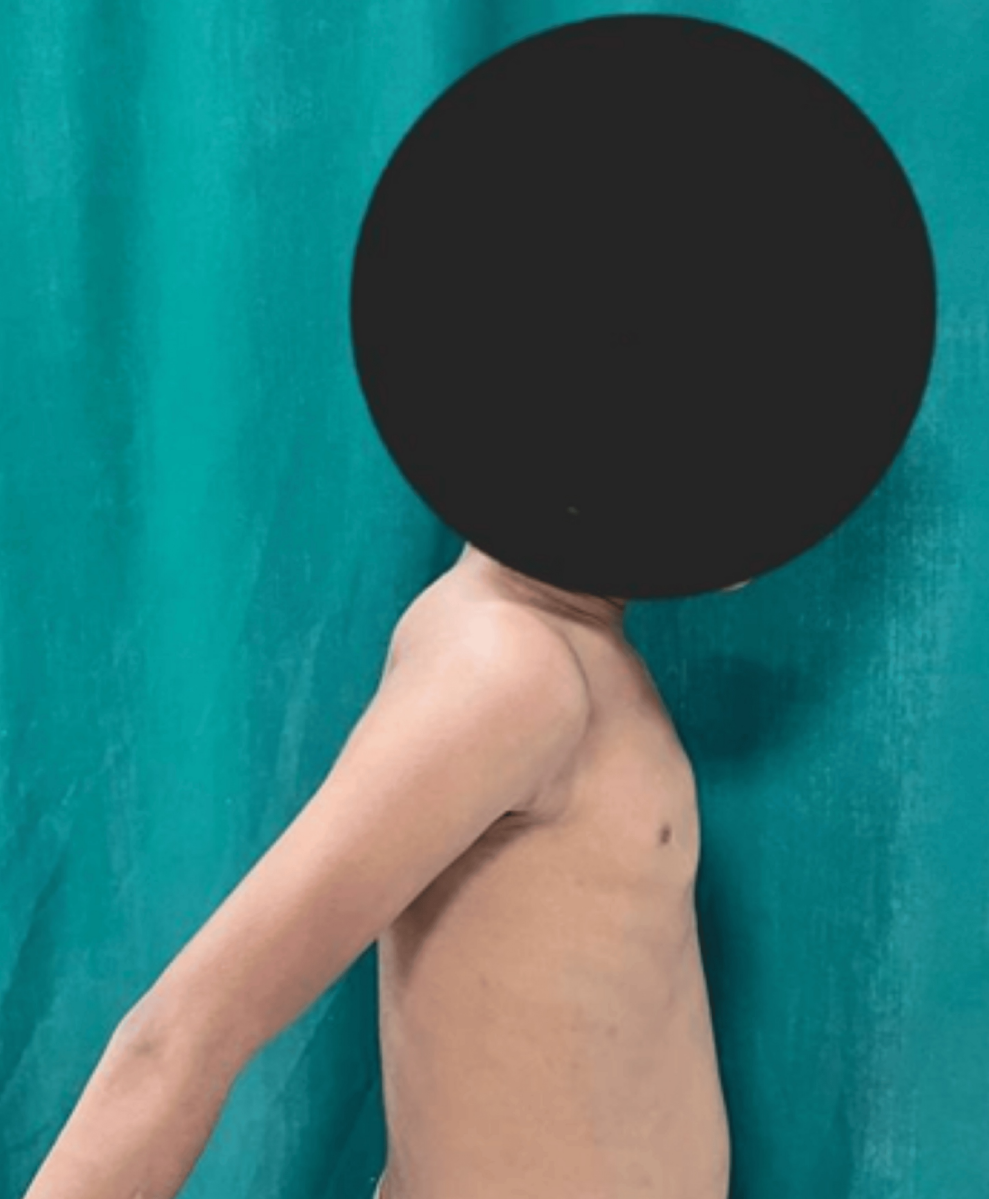
Extension of the right shoulder

A full range of motion was achieved at the three-month follow-up, as depicted: external rotation in Figure 4, abduction/forward flexion in Figure 5, and extension in Figure 6. 

## Discussion

Fractures involving the proximal humerus in the paediatric age group are rather uncommon, amounting to 0.45-2% of the upper limb fractures, and can occur in around 3% of the paediatric population [1,6].

These fractures can be managed conservatively as well as surgically. Non-operative treatment is preferred in younger age groups due to their remarkable remodelling capacity and in minimally displaced fractures [7]. Surgical management is preferred in older age groups and moderate to severely displaced fractures; closed reduction and internal fixation using K-wires is a reliable method of fixation for these fractures. It has the advantages of being less invasive, with minimal damage to the soft tissues and reduced risk of avascular necrosis of the humeral head [8]. Other surgical options include elastic stable intramedullary nailing, percutaneous fixation with cannulated screws, and open reduction and fixation using a plate; however, these methods have disadvantages such as increased surgical time, more soft tissue damage, increased risk of injury to the neurovascular structures, and increased risk of damage to the epiphysis, which may ultimately lead to avascular necrosis [9]. The major problems associated with the percutaneous pinning method are the risk of pin migration and infections of the pin tract, which should be carefully monitored.

Closed reduction of the fracture can be achieved easily in the paediatric age group under fluoroscopic guidance. The pins are usually removed around three to four weeks postoperatively, and the joint is mobilized progressively. Percutaneous pinning is a reliable method for ensuring a pain-free and near-total range of motion in the paediatric age group. Advantages of percutaneous pinning of proximal humerus fracture in children include minimal soft tissue dissection, which leads to reduced surgical time and a lower risk of damage to neurovascular structures. Additionally, this technique reduces the risk of damage to the epiphysis and avascular necrosis of the humeral head. However, there are some disadvantages, such as the potential for K-wire migration and pin tract site infection. There is also a risk of increased shoulder stiffness post-surgery, and limb length discrepancy may occur over the long term, especially if there is damage to the physis.

## Conclusions

Fractures involving the proximal humerus in the paediatric age group usually occur as a result of a fall or high-velocity trauma. Management of these fractures is dependent on various factors, such as the age of the patient, displacement of the fracture fragments, and the capacity to remodel. Managing the fracture conservatively is usually adequate in younger age groups, especially when the fracture is less displaced. Surgical intervention is required if the fracture is moderate to severely displaced. Closed reduction and internal fixation using K-wires is a good treatment modality for proximal humerus fractures in the paediatric age group. Percutaneous pinning of these fractures allows us to fix the fracture with minimal damage to the soft tissues and neurovascular structures while giving us a near-normal range of motion of the shoulder joint.
